# Supplementary material for: Covid-19 and cardiovascular disease in a total population-study of long-term effects, social factors and Covid-19-vaccination
Source: Nat Commun. 2025 Nov 18;16:10115. doi: 10.1038/s41467-025-66270-1 (PMC12627555; doi:10.1038/s41467-025-66270-1)
Supplement: Supplementary file 1 — Supplementary Information [file 41467_2025_66270_MOESM1_ESM.pdf]

Covid-19 and cardiovascular disease in a total population-study of long-term effects, social factors and Covid-19-vaccination

Malin Spetz[1,2*], Yvonne Natt och Dag[1,3], Huiqi Li[1], Fredrik Nyberg[1], Maria Rosvall[1,3].

1. School of Public Health and Community Medicine, Institute of Medicine, Sahlgrenska Academy, University of Gothenburg, Gothenburg, Sweden
2. Department of strategic healthcare development, Head office, Region Västra Götaland, Gothenburg, Sweden
3. Department of Social Medicine, FoUUI, Regionhälsan, Region Västra Götaland, Gothenburg, Sweden

* Corresponding author:
Malin Spetz
E-mail: malin.spetz@gu.se
Telephone: +4631786 00 00
Postal address: School of Public Health and Community Medicine, Institute of Medicine, Sahlgrenska Academy, University of Gothenburg
Box 453
405 30 Gothenburg
SWEDEN

Contents of this file:

Supplementary Tables 1-17

Supplementary Figure 1

Supplementary table 1 Risk of cardiovascular disease following Covid-19 also including data regarding cause-specific deaths corresponding to the studied outcomes from the National Cause of Death Register (NCDR) This table shows the risk of various cardiovascular disease (CVD) outcomes related to Covid-19 [hazard ratios (HRs) with 95% confidence intervals (CI)] in a cohort of individuals in Sweden aged 40-75 years (n=4,095,414) followed from 1 January 2020 to 31 December 2021, also including data regarding cause-specific deaths corresponding to the studied outcomes from the National Cause of Death Register (NCDR), from three different Cox regression models with different adjustments.

**Incident CVD outcomes**	**Covid-19**[b]	**Model 1**[c]	**Model 2**[e]	**Model 3**[f]
		HR (95% CI)	HR (95% CI)	HR (95% CI)
**Ischemic stroke**	No	1.00[d]	1.00[d]	1.00[d]
(n= 15 569[a])	Yes	1.41 (1.31-1.52)	1.46 (1.36-1.57)	1.43 (1.32-1.54)
**Intracerebral hemorrhage**	No	1.00[d]	1.00[d]	1.00[d]
(n= 3 315[a])	Yes	1.46 (1.25-1.70)	1.51 (1.30-1.76)	1.48 (1.27-1.73)
**Cerebrovascular disease**	No	1.00[d]	1.00[d]	1.00[d]
(n= 25 938[a])	Yes	1.47 (1.39-1.56)	1.53 (1.44-1.61)	1.49 (1.41-1.58)
**Acute myocardial infarction**	No	1.00[d]	1.00[d]	1.00[d]
(n= 19 513[a])	Yes	1.26 (1.18-1.35)	1.27 (1.18-1.35)	1.24 (1.16-1.33)
**Ischemic heart disease**	No	1.00[d]	1.00[d]	1.00[d]
(n= 43 548[a])	Yes	1.38 (1.32-1.44)	1.37 (1.31-1.43)	1.34 (1.28-1.40)
**Cardiomyopathy**	No	1.00[d]	1.00[d]	1.00[d]
(n= 3 632[a])	Yes	1.37 (1.19-1.57)	1.43 (1.25-1.64)	1.41 (1.23-1.63)
**Heart failure**	No	1.00[d]	1.00[d]	1.00[d]
(n= 21 756[a])	Yes	1.59 (1.50-1.69)	1.67 (1.57-1.77)	1.57 (1.48-1.67)
**Deep venous thrombosis**	No	1.00[d]	1.00[d]	1.00[d]
(n= 14 237[a])	Yes	1.74 (1.63-1.85)	1.80 (1.69-1.92)	1.78 (1.67-1.90)
**Pulmonary embolism**	No	1.00[d]	1.00[d]	1.00[d]
(n= 13 639[a])	Yes	4.25 (4.03-4.47)	4.39 (4.17-4.62)	4.24 (4.02-4.47)

a. Number of events
b. Defined as a time-varying exposure
c. Model 1: Adjusted for age
d. Reference group
e. Model 2: Adjusted for age, sex, country of birth, income and education
f. Model 3: Adjusted for age, sex, country of birth, income, education, comorbidities and vaccination against Covid-19

Supplementary Table 2 Risk of all cause and cardiovascular mortality following Covid-19. This table shows the risk of all cause mortality and cardiovascular mortality related to Covid-19 [hazard ratios (HRs) with 95% confidence intervals (CI)] in a cohort of individuals in Sweden aged 40-75 years (n=4,095,414) followed from 1 January 2020 to 31 December 2021, from three different Cox regression models with different adjustments.

**Mortality**	**Covid-19[c]**	**Model 1[d]**	**Model 2[f]**	**Model 3[g]**
		HR (95% CI)	HR (95% CI)	HR (95% CI)
**All cause**	No	1.00[e]	1.00[e]	1.00[e]
(n[a]=41 503; IR[b] =510.9 (95% CI:506.0-515.9))	Yes	3.20 (3.09-3.31)	3.46 (3.34-3.58)	3.19 (3.08-3.30)
**Cardiovascular disease**	No	1.00[e]	1.00[e]	1.00[e]
(n[a]=16 374; IR[b] =201.6 (95% CI:198.5-204.7))	Yes	3.45 (3.27-3.64)	3.78 (3.58-3.99)	3.46 (3.28-3.65)
**Ischemic heart disease**	No	1.00[e]	1.00[e]	1.00[e]
(n[a]=5 125; IR[b] =63.1 (95% CI:61.4-64.8))	Yes	1.69 (1.49-1.91)	1.89 (1.67-2.13)	1.76 (1.56-1.99)

a. Number of events during follow-up
b. IR: Incidence rate per 100 000 person-years
c. Defined as a time-varying exposure
d. Model 1: Adjusted for age.
e. Reference group
f. Model 2: Adjusted for age, sex, country of birth, income and education
g. Model 3: Adjusted for age, sex, country of birth, income, education, comorbidities and vaccination against Covid-19

Supplementary table 3 Risk of cardiovascular disease following Covid-19 among those without prior comorbidities. This table outlines the risk of various cardiovascular disease (CVD) outcomes related to Covid-19 [hazard ratios (HRs) with 95% confidence intervals (CI)] in a cohort of individuals in Sweden aged 40-75 years without prior comorbidities (n=3,214,001) followed from 1 January 2020 to 31 December 2021, from three different Cox regression models with different adjustments.

**Incident CVD outcomes**	**Covid-19[b]**	**Model 1[c]**	**Model 2[e]**	**Model 3[f]**
		HR (95% CI)	HR (95% CI)	HR (95% CI)
**Ischemic stroke** (n= 10 091[a])	No	1.00[d]	1.00[d]	1.00[d]
	Yes	1.29 (1.17-1.42)	1.34 (1.22-1.47)	1.33 (1.21-1.47)
**Intracerebral hemorrhage** (n=2 085 [a])	No	1.00[d]	1.00[d]	1.00[d]
	Yes	1.24 (1.02-1.52)	1.29 (1.06-1.58)	1.29 (1.06-1.58)
**Cerebrovascular disease** (n=16 421[a])	No	1.00[d]	1.00[d]	1.00[d]
	Yes	1.32 (1.22-1.42)	1.37 (1.27-1.47)	1.36 (1.27-1.47)
**Acute myocardial infarction** (n=12 209 [a])	No	1.00[d]	1.00[d]	1.00[d]
	Yes	1.19 (1.10-1.30)	1.19 (1.09-1.30)	1.19 (1.09-1.30)
**Ischemic heart disease** (n= 26 561[a])	No	1.00[d]	1.00[d]	1.00[d]
	Yes	1.36 (1.28-1.44)	1.34 (1.26-1.42)	1.34 (1.27-1.42)
**Cardiomyopathy** (n=2 318 [a])	No	1.00[d]	1.00[d]	1.00[d]
	Yes	1.42 (1.20-1.68)	1.47 (1.24-1.74)	1.47 (1.24-1.74)
**Heart failure** (n=11 682 [a])	No	1.00[d]	1.00[d]	1.00[d]
	Yes	1.34 (1.23-1.46)	1.41 (1.29-1.54)	1.40 (1.29-1.53)
**Deep venous thrombosis** (n=9 363 [a])	No	1.00[d]	1.00[d]	1.00[d]
	Yes	1.73 (1.60-1.87)	1.78 (1.65-1.92)	1.77 (1.64-1.92)
**Pulmonary embolism** (n=7 912 [a])	No	1.00[d]	1.00[d]	1.00[d]
	Yes	4.82 (4.52-5.15)	4.94 (4.63-5.27)	4.88 (4.57-5.21)

a. Number of events during follow-up
b. Defined as a time-varying exposure
c. Model 1: Adjusted for age
d. Reference group
e. Model 2: Adjusted for age, sex, country of birth, income and education
f. Model 3: Adjusted for age, sex, country of birth, income, education, and vaccination against Covid-19

Supplementary table 4 Risk of all cause and cardiovascular mortality following Covid-19 among those without prior comorbidities. This table shows the risk of all cause mortality and cardiovascular mortality related to Covid-19 infection [hazard ratios (HRs) with 95% confidence intervals (CI)] in a cohort of individuals in Sweden aged 40-75 years without prior comorbidities (n=3,214,001) followed from 1 January 2020 to 31 December 2021, from three different Cox regression models with different adjustments.

**Mortality**	**Covid-19**[b]	**Model 1**[c]	**Model 2**[e]	**Model 3**[f]
		HR (95% CI)	HR (95% CI)	HR (95% CI)
**All cause**	No	1.00[d]	1.00[d]	1.00[d]
(n=18 668[a])	Yes	2.81 (2.68-2.96)	3.09 (2.93-3.25)	3.04 (2.89-3.20)
**Cardiovascular disease**	No	1.00[d]	1.00[d]	1.00[d]
(n=8 500[a])	Yes	2.82 (2.61-3.05)	3.12 (2.88-3.37)	3.07 (2.84-3.32)
**Ischemic heart disease**	No	1.00[d]	1.00[d]	1.00[d]
(n=3 111[a])	Yes	1.39 (1.17-1.65)	1.56 (1.32-1.86)	1.52 (1.28-1.81)

a. Number of events during follow-up
b. Defined as a time-varying exposure
c. Model 1: Adjusted for age.
d. Reference group
e. Model 2: Adjusted for age, sex, country of birth, income and education
f. Model 3: Adjusted for age, sex, country of birth, income, education, and vaccination against Covid-19

Supplementary table 5 Risk of cardiovascular disease following Covid-19 in a cohort of individuals in Sweden aged 18-39 years. This table shows the risk of various cardiovascular disease (CVD) outcomes related to Covid-19 infection [hazard ratios (HRs) with 95% confidence intervals (CI)] in a cohort of individuals in Sweden aged 18-39 years (n=2,905,382) followed from 1 January 2020 to 31 December 2021, from three different Cox regression models with different adjustments.

**Incident CVD outcomes**	**Covid-19[b]**	**Model 1[c]**	**Model 2[e]**	**Model 3[f]**
		HR (95% CI)	HR (95% CI)	HR (95% CI)
**Ischemic stroke** (n=589[a])	No	1.00[d]	1.00[d]	1.00[d]
	Yes	1.15 (0.84-1.56)	1.17 (0.86-1.59)	1.16 (0.85-1.58)
**Intracerebral hemorrhage** (n=248[a])	No	1.00[d]	1.00[d]	1.00[d]
	Yes	1.31 (0.82-2.10)	1.38 (0.86-2.20)	1.35 (0.85-2.16)
**Cerebrovascular disease** (n=1 377[a])	No	1.00[d]	1.00[d]	1.00[d]
	Yes	1.21 (0.99-1.48)	1.23 (1.01-1.50)	1.22 (1.00-1.49)
**Acute myocardial infarction** (n= 319[a])	No	1.00[d]	1.00[d]	1.00[d]
	Yes	1.01 (0.67-1.51)	1.04 (0.70-1.56)	1.03 (0.69-1.54)
**Ischemic heart disease** (n=528[a])	No	1.00[d]	1.00[d]	1.00[d]
	Yes	1.07 (0.79-1.46)	1.11 (0.81-1.51)	1.10 (0.80-1.50)
**Cardiomyopathy** (n=458[a])	No	1.00[d]	1.00[d]	1.00[d]
	Yes	0.89 (0.61-1.30)	0.93 (0.63-1.36)	0.93 (0.63-1.36)
**Heart failure** (n=769[a])	No	1.00[d]	1.00[d]	1.00[d]
	Yes	1.49 (1.17-1.89)	1.57 (1.23-2.00)	1.56 (1.22-1.98)
**Deep venous thrombosis** (n=2 645[a])	No	1.00[d]	1.00[d]	1.00[d]
	Yes	1.34 (1.17-1.54)	1.34 (1.17-1.54)	1.33 (1.16-1.53)
**Pulmonary embolism** (n=1 610[a])	No	1.00[d]	1.00[d]	1.00[d]
	Yes	3.52 (3.07-4.03)	3.57 (3.11-4.08)	3.55 (3.10-4.07)

a. Number of events during follow-up
b. Defined as a time-varying exposure
c. Model 1: Adjusted for age
d. Reference group
e. Model 2: Adjusted for age, sex, country of birth, income and education
f. Model 3: Adjusted for age, sex, country of birth, income, education, comorbidities and vaccination against Covid-19

Supplementary table 6 Risk of all cause and cardiovascular mortality following Covid-19 in a cohort of individuals in Sweden aged 18-39 years. This table shows the risk of all cause mortality and cardiovascular mortality related to Covid-19 infection [hazard ratios (HRs) with 95% confidence intervals (CI)] in a cohort of individuals in Sweden aged 18-39 years (n=2,905,382) followed from 1 January 2020 to 31 December 2021, from three different Cox regression models with different adjustments.

**Mortality**	**Covid-19**[b]	**Model 1**[c]	**Model 2**[e]	**Model 3**[f]
		HR (95% CI)	HR (95% CI)	HR (95% CI)
**All cause** (n=2 802[a])	No	1.00[d]	1.00[d]	1.00[d]
	Yes	1.10 (0.95-1.27)	1.23 (1.07-1.42)	1.27 (1.10-1.46)
**Cardiovascular disease** (n=369[a])	No	1.00[d]	1.00[d]	1.00[d]
	Yes	1.93 (1.39-2.67)	2.22 (1.60-3.08)	2.22 (1.60-3.08)
**Ischemic heart disease** (n=48[a])	No	1.00[d]	1.00[d]	1.00[d]
	Yes	1.42 (0.54-3.74)	1.65 (0.63-4.34)	1.64 (0.62-4.31)

a. Number of events during follow-up
b. Defined as a time-varying exposure
c. Model 1: Adjusted for age.
d. Reference group
e. Model 2: Adjusted for age, sex, country of birth, income and education
f. Model 3: Adjusted for age, sex, country of birth, income, education, comorbidities and vaccination against Covid-19

Supplementary table 7 Risk of cardiovascular disease following Covid-19 in a cohort of individuals in Sweden aged > 75 years. This table shows the risk of various cardiovascular disease (CVD) outcomes related to Covid-19 infection [hazard ratios (HRs) with 95% confidence intervals (CI)] in a cohort of individuals in Sweden aged > 75 years (n=629,933) followed from 1 January 2020 to 31 December 2021, from three different Cox regression models with different adjustments.

**Incident CVD outcomes**	**Covid-19[b]**	**Model 1[c]**	**Model 2[e]**	**Model 3[f]**
		HR (95% CI)	HR (95% CI)	HR (95% CI)
**Ischemic stroke** (n=13 102[a])	No	1.00[d]	1.00[d]	1.00[d]
	Yes	1.26 (1.14-1.40)	1.28 (1.15-1.42)	1.25 (1.12-1.39)
**Intracerebral hemorrhage** (n=2 298[a])	No	1.00[d]	1.00[d]	1.00[d]
	Yes	1.27 (0.99-1.63)	1.30 (1.01-1.66)	1.27 (0.99-1.62)
**Cerebrovascular disease** (n=20 834[a])	No	1.00[d]	1.00[d]	1.00[d]
	Yes	1.46 (1.35-1.58)	1.48 (1.37-1.60)	1.44 (1.33-1.56)
**Acute myocardial infarction** (n= 9 213[a])	No	1.00[d]	1.00[d]	1.00[d]
	Yes	1.27 (1.12-1.44)	1.29 (1.14-1.47)	1.25 (1.11-1.42)
**Ischemic heart disease** (n=24 985[a])	No	1.00[d]	1.00[d]	1.00[d]
	Yes	1.38 (1.28-1.48)	1.41 (1.30-1.51)	1.38 (1.28-1.48)
**Cardiomyopathy** (n=1 006[a])	No	1.00[d]	1.00[d]	1.00[d]
	Yes	1.10 (0.72-1.66)	1.11 (0.73-1.68)	1.08 (0.71-1.64)
**Heart failure** (n=28 865[a])	No	1.00[d]	1.00[d]	1.00[d]
	Yes	1.59 (1.49-1.69)	1.61 (1.51-1.71)	1.51 (1.42-1.61)
**Deep venous thrombosis** (n=4 750[a])	No	1.00[d]	1.00[d]	1.00[d]
	Yes	1.79 (1.54-2.09)	1.82 (1.56-2.13)	1.79 (1.54-2.09)
**Pulmonary embolism** (n=6 041[a])	No	1.00[d]	1.00[d]	1.00[d]
	Yes	3.61 (3.27-3.98)	3.63 (3.29-4.01)	3.48 (3.15-3.85)

a. Number of events during follow-up
b. Defined as a time-varying exposure
c. Model 1: Adjusted for age
d. Reference group
e. Model 2: Adjusted for age, sex, country of birth, income and education
f. Model 3: Adjusted for age, sex, country of birth, income, education, comorbidities and vaccination against Covid-19

Supplementary table 8 Risk of all cause and cardiovascular mortality following Covid-19 in a cohort of individuals in Sweden aged >75 years. This table shows the risk of all cause mortality and cardiovascular mortality related to Covid-19 infection [hazard ratios (HRs) with 95% confidence intervals (CI)] in a cohort of individuals in Sweden aged > 75 years (n=629,933) followed from 1 January 2020 to 31 December 2021, from three different Cox regression models with different adjustments.

Mortality	Covid-19[b]	Model 1[c]	Model 2[e]	Model 3[f]
		HR (95% CI)	HR (95% CI)	HR (95% CI)
**All cause** (n=71 992[a])	No	1.00[d]	1.00[d]	1.00[d]
	Yes	5.77 (5.64-5.90)	5.87 (5.73-6.00)	5.51 (5.38-5.63)
**Cardiovascular disease** (n=39 785[a])	No	1.00[d]	1.00[d]	1.00[d]
	Yes	5.15 (5.00-5.32)	5.27 (5.11-5.44)	4.95 (4.79-5.10)
**Ischemic heart disease** (n=7 985[a])	No	1.00[d]	1.00[d]	1.00[d]
	Yes	3.59 (3.32-3.88)	3.72 (3.44-4.03)	3.61 (3.33-3.90)

a. Number of events during follow-up
b. Defined as a time-varying exposure
c. Model 1: Adjusted for age.
d. Reference group
e. Model 2: Adjusted for age, sex, country of birth, income and education
f. Model 3: Adjusted for age, sex, country of birth, income, education, comorbidities and vaccination against Covid-19

Supplementary table 9 Risk of cardiovascular disease and mortality following Covid-19 starting follow-up 1 March 2020

This table show the risk of various cardiovascular disease (CVD) and mortality outcomes related to Covid-19 [hazard ratios (HRs) with 95% confidence intervals (CI)] in a cohort of individuals in Sweden aged 40-75 years (n= 4,092,728) followed from 1 March 2020 to 31 December 2021, from a fully adjusted Cox regression model (model 3).

Incident CVD and mortality outcomes	Covid-19[b]	Model 3[c]
		HR (95% CI)
**Ischemic stroke**	No	1.00d[d]
(n= 14 026[a])	Yes	1.38 (1.27-1.48)
**Intracerebral hemorrhage**	No	1.00[d]
(n= 2 866[a])	Yes	1.44 (1.23-1.69)
**Cerebrovascular disease**	No	1.00[d]
(n= 23 065[a])	Yes	1.44 (1.36-1.53)
**Acute myocardial infarction**	No	1.00[d]
(n= 16 146[a])	Yes	1.22 (1.14-1.31)
**Ischemic heart disease**	No	1.00[d]
(n= 36 084[a])	Yes	1.34 (1.28-1.41)
**Cardiomyopathy**	No	1.00[d]
(n= 3 185[a])	Yes	1.39 (1.20-1.60)
**Heart failure**	No	1.00[d]
(n= 19 117[a])	Yes	1.54 (1.45-1.64)
**Deep venous thrombosis**	No	1.00[d]
(n= 12 912[a])	Yes	1.77 (1.66-1.89)
**Pulmonary embolism**	No	1.00[d]
(n= 12 111[a])	Yes	4.32 (4.10-4.56)
**All cause mortality**	No	1.00[d]
(n= 38 810[a])	Yes	3.21 (3.10-3.32)
**Cardiovascular disease mortality**	No	1.00[d]
(n= 15 398[a])	Yes	3.47 (3.29-3.66)
**Ischemic heart disease mortality**	No	1.00[d]
(n= 4 801[a])	Yes	1.77 (1.56-2.00)

a. Number of events during follow-up
b. Defined as a time-varying exposure
c. Model 3: Adjusted for age, sex, country of birth, income, education, comorbidities and vaccination against Covid-19
d. Reference group

Supplementary table 10 Risk of cardiovascular disease and mortality outcomes following Covid-19 excluding individuals who immigrated during the five-year lookback period

This table shows the risk of various cardiovascular disease (CVD) and mortality outcomes related to Covid-19 [hazard ratios (HRs) with 95% confidence intervals (CI)] in a cohort of individuals in Sweden aged 40-75 years (n=3,963,612) followed from 1 January 2020 to 31 December 2021, excluding individuals who immigrated during the five-year lookback period, from three different Cox regression models with different adjustments.

Incident CVD and mortality outcomes	Covid-19[b]	Model 1[c]	Model 2[e]	Model 3[f]
		HR (95% CI)	HR (95% CI)	HR (95% CI)
**Ischemic stroke** (n= 14 892[a])	No	1.00[d]	1.00[d]	1.00[d]
	Yes	1.36 (1.26-1.47)	1.40 (1.30-1.52)	1.37 (1.27-1.48)
**Intracerebral hemorrhage** (n= 3 034[a])	No	1.00[d]	1.00[d]	1.00[d]
	Yes	1.39 (1.18-1.63)	1.43 (1.22-1.69)	1.40 (1.19-1.65)
**Cerebrovascular disease** (n= 24 654[a])	No	1.00[d]	1.00[d]	1.00[d]
	Yes	1.42 (1.34-1.50)	1.47 (1.38-1.56)	1.44 (1.35-1.52)
**Acute myocardial infarction** (n= 17 112[a])	No	1.00[d]	1.00[d]	1.00[d]
	Yes	1.24 (1.15-1.33)	1.23 (1.14-1.32)	1.21 (1.12-1.30)
**Ischemic heart disease** (n= 38 562[a])	No	1.00[d]	1.00[d]	1.00[d]
	Yes	1.38 (1.32-1.45)	1.36 (1.30-1.43)	1.33 (1.27-1.40)
**Cardiomyopathy** (n= 3 349[a])	No	1.00[d]	1.00[d]	1.00[d]
	Yes	1.35 (1.17-1.56)	1.40 (1.21-1.62)	1.38 (1.20-1.60)
**Heart failure** (n= 20 349[a])	No	1.00[d]	1.00[d]	1.00[d]
	Yes	1.55 (1.46-1.66)	1.63 (1.53-1.73)	1.54 (1.44-1.64)
**Deep venous thrombosis** (n= 13 919[a])	No	1.00[d]	1.00[d]	1.00[d]
	Yes	1.71 (1.60-1.82)	1.77 (1.66-1.89)	1.74 (1.63-1.86)
**Pulmonary embolism** (n= 12 834[a])	No	1.00[d]	1.00[d]	1.00[d]
	Yes	4.30 (4.07-4.53)	4.42 (4.19-4.66)	4.27 (4.05-4.51)
**All cause mortality** (n= 40 758[a])	No	1.00[d]	1.00[d]	1.00[d]
	Yes	3.18 (3.07-3.29)	3.43 (3.32-3.55)	3.17 (3.06-3.28)
**Cardiovascular disease mortality** (n= 16 128[a])	No	1.00[d]	1.00[d]	1.00[d]
	Yes	3.42 (3.24-3.61)	3.75 (3.55-3.96)	3.44 (3.26-3.63)
**Ischemic heart disease mortality** (n= 5 049[a])	No	1.00[d]	1.00[d]	1.00[d]
	Yes	1.70 (1.50-1.92)	1.89 (1.67-2.14)	1.77 (1.56-2.00)

a. Number of events during follow-up
b. Defined as a time-varying exposure
c. Model 1: Adjusted for age
d. Reference group
e. Model 2: Adjusted for age, sex, country of birth, income and education
f. Model 3: Adjusted for age, sex, country of birth, income, education, comorbidities and vaccination against Covid-19

Supplementary Table 11 Risk of cardiovascular disease following Covid-19 by severity. This table shows the risk of various cardiovascular disease (CVD) outcomes related to Covid-19 [hazard ratios (HRs) with 95% confidence intervals (CI)] in a cohort of individuals in Sweden aged 40-75 years (n=4,095,414) followed from 1 January 2020 to 31 December 2021, from three different Cox regression models with different adjustments, by hospitalisation due to Covid-19.

**Incident CVD outcomes**	**Covid-19**[b]	**Model 1**[c]	**Model 2**[e]	**Model 3**[f]
		HR (95% CI)	HR (95% CI)	HR (95% CI)
**Ischemic stroke** (n=15 175[a])	No	1.00[d]	1.00[d]	1.00[d]
	Yes, non hospitalised	1.01 (0.92-1.11)	1.05 (0.96-1.16)	1.04 (0.95-1.15)
	Yes, hospitalised	4.01 (3.53-4.54)	3.74 (3.30-4.24)	3.35 (2.95 -3.80)
**Intracerebral hemorrhage** (n=3 092[a])	No	1.00[d]	1.00[d]	1.00[d]
	Yes, non hospitalised	0.88 (0.72-1.08)	0.93 (0.75-1.14)	0.92 (0.75-1.13)
	Yes, hospitalised	5.88 (4.67-7.41)	5.42 (4.30-6.83)	4.91 (3.89-6.19)
**Cerebrovascular disease** (n=25 117[a])	No	1.00[d]	1.00[d]	1.00[d]
	Yes, non hospitalised	0.99 (0.92-1.06)	1.03 (0.96-1.11)	1.03 (0.96-1.11)
	Yes, hospitalised	4.79 (4.38-5.25)	4.50 (4.11-4.93)	4.01 (3.66-4.40)
**Acute myocardial infarction** (n=17 589[a])	No	1.00[d]	1.00[d]	1.00[d]
	Yes, non hospitalised	1.03 (0.95-1.12)	1.05 (0.97-1.14)	1.04 (0.96-1.13)
	Yes, hospitalised	3.10 (2.72-3.54)	2.52 (2.21-2.88)	2.31 (2.02-2.64)
**Ischemic heart disease** (n=39 665[a])	No	1.00[d]	1.00[d]	1.00[d]
	Yes, non hospitalised	1.06 (1.00-1.12)	1.07 (1.02-1.14)	1.07 (1.01-1.13)
	Yes, hospitalised	4.05 (3.74-4.38)	3.24 (2.99-3.50)	2.92 (2.70-3.16)
**Cardiomyopathy** (n=3 431 [a])	No	1.00[d]	1.00[d]	1.00[d]
	Yes, non hospitalised	1.01 (0.85-1.19)	1.05 (0.88-1.24)	1.05 (0.88-1.24)
	Yes, hospitalised	4.77 (3.75-6.06)	4.73 (3.71-6.01)	4.25 (3.33-5.41)
**Heart failure** (n=20 716[a])	No	1.00[d]	1.00[d]	1.00[d]
	Yes, non hospitalised	0.96 (0.88-1.04)	1.02 (0.94-1.11)	1.00 (0.92-1.09)
	Yes, hospitalised	5.89 (5.40-6.44)	5.40 (4.94-5.91)	4.19 (3.83-4.58)
**Deep venous thrombosis** (n=14 135[a])	No	1.00[d]	1.00[d]	1.00[d]
	Yes, non hospitalised	1.37 (1.27-1.48)	1.41 (1.31-1.52)	1.41 (1.31-1.52)
	Yes, hospitalised	5.33 (4.75-5.99)	5.78 (5.14-6.49)	5.20 (4.62-5.84)
**Pulmonary embolism** (n=13 080[a])	No	1.00[d]	1.00[d]	1.00[d]
	Yes, non hospitalised	1.75 (1.61-1.89)	1.80 (1.67-1.95)	1.78 (1.64-1.92)
	Yes, hospitalised	25.92 (24.37-27.56)	26.33 (24.72-28.03)	22.94 (21.52-24.44)

a. Number of events during follow-up
b. Defined as a time-varying exposure
c. Model 1: Adjusted for age
d. Reference group
e. Model 2: Adjusted for age, sex, country of birth, income and education
f. Model 3: Adjusted for age, sex, country of birth, income, education, comorbidities and vaccination against Covid-19

Supplementary Table 12 Risk of all cause and cardiovascular mortality following Covid-19 by severity. This table shows the risk of all cause mortality and cardiovascular mortality related to Covid-19 [hazard ratios (HRs) with 95% confidence intervals (CI)] in a cohort of individuals in Sweden aged 40-75 years (n=4,095,414) followed from 1 January 2020 to 31 December 2021, from three different Cox regression models with different adjustments, by hospitalisation due to Covid-19.

**Mortality**	**Covid-19**[b]	**Model 1**[c]	**Model 2**[e]	**Model 3**[f]
		HR (95% CI)	HR (95% CI)	HR (95% CI)
**All cause** (n=41 503[a])	No	1.00[d]	1.00[d]	1.00[d]
	Yes, non hospitalised	1.39 (1.32-1.46)	1.54 (1.47-1.62)	1.49 (1.42-1.57)
	Yes, hospitalised	15.20 (14.59-15.84)	14.43 (13.84-15.04)	10.77 (10.33-11.24)
**Cardiovascular disease** (n=16 374[a])	No	1.00[d]	1.00[d]	1.00[d]
	Yes, non hospitalised	1.42 (1.31-1.54)	1.61 (1.48-1.75)	1.56 (1.43-1.69)
	Yes, hospitalised	15.80 (14.82-16.84)	14.81 (13.88-15.80)	11.04 (10.34-11.79)
**Ischemic heart disease** (n=5 125[a])	No	1.00[d]	1.00[d]	1.00[d]
	Yes, non hospitalised	0.97 (0.82-1.15)	1.12 (0.95-1.33)	1.09 (0.92-1.29)
	Yes, hospitalised	6.40 (5.41-7.58)	5.87 (4.95-6.95)	4.65 (3.92-5.51)

a. Number of events during follow-up
b. Defined as a time-varying exposure
c. Model 1: Adjusted for age
d. Reference group
e. Model 2: Adjusted for age, sex, country of birth, income and education
f. Model 3: Adjusted for age, sex, country of birth, income, education, comorbidities and vaccination against Covid-19

Supplementary Table 13 Risk of cardiovascular disease and mortality following Covid-19 by severity, stratified by pandemic waves. This table shows the risk of various cardiovascular disease (CVD) and mortality outcomes related to Covid-19 [hazard ratios (HRs) with 95% confidence intervals (CI)] in a cohort of individuals in Sweden aged 40-75 years (n=4,095,414) followed from 1 January 2020 to 31 December 2021, from fully adjusted Cox regression models, by hospitalisation, stratified by pandemic waves.

		**Pandemic wave 1**	**Pandemic wave 2**	**Pandemic wave 3**	**Pandemic wave 4**
**Outcome**	**Covid-19[a]**	HR[b] (95% CI)	HR[b] (95% CI)	HR[b] (95% CI)	HR[b] (95% CI)
**Ischemic stroke**	No	1.00[c]	1.00[c]	1.00[c]	1.00[c]
	Yes, non hospitalised	1.07 (0.59-1.94)	1.33 (1.02-1.75)	1.00 (0.85-1.18)	1.02 (0.90-1.16)
	Yes, hospitalised	5.51 (4.00-7.60)	5.54 (4.19-7.34)	4.24 (3.47-5.16)	1.68 (1.29-2.18)
**Intracerebral hemorrhage**	No	1.00[c]	1.00[c]	1.00[c]	1.00[c]
	Yes, non hospitalised	- [d]	- [d]	0.95 (0.67-1.34)	1.03 (0.78-1.36)
	Yes, hospitalised	15.89 (10.44-24.19)	5.11 (2.71-9.62)	5.00 (3.30-7.56)	2.57 (1.60-4.12)
**Cerebrovascular disease**	No	1.00[c]	1.00[c]	1.00[c]	1.00[c]
	Yes, non hospitalised	1.04 (0.66-1.66)	1.13 (0.90-1.41)	1.01 (0.89-1.14)	1.02 (0.92-1.13)
	Yes, hospitalised	8.26 (6.71-10.16)	7.05 (5.81-8.55)	4.76 (4.10-5.53)	1.77 (1.45-2.16)
**Acute myocardial infarction**	No	1.00[c]	1.00[c]	1.00[c]	1.00[c]
	Yes, non hospitalised	1.23 (0.75.2.04)	0.98 (0.75-1.28)	1.06 (0.92-1.22)	1.02 (0.92-1.15)
	Yes, hospitalised	3.59 (2.53-5.09)	3.44 (2.53-4.65)	2.44 (1.93-3.07)	1.65 (1.31-2.07)
**Ischemic heart disease**	No	1.00[c]	1.00[c]	1.00[c]	1.00[c]
	Yes, non hospitalised	1.06 (0.73-1.54)	0.98 (0.81-1.18)	1.09 (1.00-1.20)	1.05 (0.97-1.13)
	Yes, hospitalised	3.69 (2.94-4.63)	5.59 (4.76-6.57)	3.37 (2.96-3.85)	1.66 (1.42-1.94)
**Cardiomyopathy**	No	1.00[c]	1.00[c]	1.00[c]	1.00[c]
	Yes, non hospitalised	- [d]	1.21 (0.71-2.06)	1.04 (0.78-1.38)	1.07 (0.84-1.35)
	Yes, hospitalised	5.15 (2.44-10.86)	6.20 (3.48-11.07)	5.68 (3.92-8.22)	2.67 (1.71-4.18)
**Heart failure**	No	1.00[c]	1.00[c]	1.00[c]	1.00[c]
	Yes, non hospitalised	1.58 (1.02-2.46)	1.08 (0.83-1.41)	0.95 (0.82-1.09)	1.00 (0.89-1.12)
	Yes, hospitalised	7.79 (6.24-9.72)	8.49 (7.04-10.24)	4.35 (3.72-5.08)	2.41 (2.03-2.86)
**Deep venous thrombosis**	No	1.00[c]	1.00[c]	1.00[c]	1.00[c]
	Yes, non hospitalised	1.97 (1.31-2.98)	2.13 (1.74-2.59)	1.50 (1.33-1.70)	1.17 (1.05-1.31)
	Yes, hospitalised	15.49 (12.33-19.45)	8.05 (6.15-10.53)	5.57 (4.55-6.81)	2.25 (1.74-2.90)
**Pulmonary embolism**	No	1.00[c]	1.00[c]	1.00[c]	1.00[c]
	Yes, non hospitalised	2.74 (1.82-4.13)	3.48 (2.93-4.13)	2.23 (2.08-2.69)	0.95 (0.82-1.09)
	Yes, hospitalised	42.22 (36.39-48.98)	48.61 (42.98-54.98)	31.31 (28.32-34.62)	5.39 (4.52-6.42)
**All-cause mortality**	No	1.00[c]	1.00[c]	1.00[c]	1.00[c]
	Yes, non hospitalised	10.96 (9.68-12.41)	3.05 (2.73-3.41)	1.33 (1.21-1.46)	0.81 (0.74-0.88)
	Yes, hospitalised	33.94 (31.31-36.78)	19.70 (18.06-21.48)	11.91 (11.06-12.81)	3.14 (2.84-3.49)
**Cardiovascular disease mortality**	No	1.00[c]	1.00[c]	1.00[c]	1.00[c]
	Yes, non hospitalised	9.88 (7.95-12.29)	2.70 (2.23-3.27)	1.57 (1.36-1.81)	0.87 (0.75-1.00)
	Yes, hospitalised	37.00 (32.67-41.90)	19.40 (16.94-22.21)	12.12 (10.81-13.60)	3.07 (2.60-3.61)
**Ischemic heart disease mortality**	No	1.00[c]	1.00[c]	1.00[c]	1.00[c]
	Yes, non hospitalised	4.02 (2.22-7.29)	1.60 (1.05-2.43)	1.42 (1.09-1.85)	0.67 (0.51-0.89)
	Yes, hospitalised	11.21 (7.63 -16.48)	8.00 (5.62-11.40)	5.52 (4.13-7.37)	1.96 (1.37-2.81)

a. Defined as a time-varying exposure
b. Adjusted for age, sex, country of birth, income, education, comorbidities and vaccination against Covid-19
c. Reference group
d. Too few events (n≤5)

Supplementary Table 14 Risk of cardiovascular disease and mortality following Covid-19 by severity, stratified by predominant virus variants. This table shows the risk of various cardiovascular disease (CVD) and mortality outcomes related to Covid-19 [hazard ratios (HRs) with 95% confidence intervals (CI)] in a cohort of individuals in Sweden aged 40-75 years (n=4,095,414) followed from 1 January 2020 to 31 December 2021, from fully adjusted Cox regression models, by hospitalisation, stratified by predominant virus variants.

		**Virus variant 1 Mix**	**Virus variant 2 Alpha**	**Virus variant 3 Delta**
**Outcome**	**Covid-19[a]**	HR[b] (95% CI)	HR[b] (95% CI)	HR[b] (95% CI)
**Ischemic stroke**	No	1.00[c]	1.00[c]	1.00[c]
	Yes, non hospitalised	1.27 (1.00-1.63)	1.00 (0.85-1.18)	1.02 (0.90-1.16)
	Yes, hospitalised	5.53 (4.48-6.83)	4.24 (3.47-5.16)	1.68 (1.29-2.18)
**Intracerebral hemorrhage**	No	1.00[c]	1.00[c]	1.00[c]
	Yes, non hospitalised	- [d]	0.95 (0.67-1.34)	1.03 (0.78-1.36)
	Yes, hospitalised	9.83 (6.92-13.96)	5.00 (3.30-7.56)	2.57 (1.60-4.12)
**Cerebrovascular disease**	No	1.00[c]	1.00[c]	1.00[c]
	Yes, non hospitalised	1.11 (0.91-1.35)	1.01 (0.89-1.14)	1.02 (0.92-1.13)
	Yes, hospitalised	7.59 (6.59-8.74)	4.76 (4.10-5.53)	1.77 (1.45-2.16)
**Acute myocardial infarction**	No	1.00[c]	1.00[c]	1.00[c]
	Yes, non hospitalised	1.03 (0.81-1.30)	1.06 (0.92-1.22)	1.02 (0.92-1.15)
	Yes, hospitalised	3.49 (2.78-4.39)	2.44 (1.93-3.07)	1.65 (1.31-2.07)
**Ischemic heart disease**	No	1.00[c]	1.00[c]	1.00[c]
	Yes, non hospitalised	0.99 (0.84-1.17)	1.09 (1.00-1.20)	1.05 (0.97-1.13)
	Yes, hospitalised	4.77 (4.19-5.44)	3.37 (2.96-3.85)	1.66 (1.42-1.94)
**Cardiomyopathy**	No	1.00[c]	1.00[c]	1.00[c]
	Yes, non hospitalised	1.05 (0.63-1.75)	1.04 (0.78-1.38)	1.07 (0.84-1.35)
	Yes, hospitalised	5.80 (3.68-9.16)	5.68 (3.92-8.22)	2.67 (1.71-4.18)
**Heart failure**	No	1.00[c]	1.00[c]	1.00[c]
	Yes, non hospitalised	1.19 (0.95-1.49)	0.95 (0.82-1.09)	1.00 (0.89-1.12)
	Yes, hospitalised	8.09 (7.02-9.33)	4.35 (3.72-5.08)	2.41 (2.03-2.86)
**Deep venous thrombosis**	No	1.00[c]	1.00[c]	1.00[c]
	Yes, non hospitalised	2.11 (1.76-2.52)	1.50 (1.33-1.70)	1.17 (1.05-1.31)
	Yes, hospitalised	11.18 (9.40-13.31)	5.57 (4.55-6.81)	2.25 (1.74-2.90)
**Pulmonary embolism**	No	1.00[c]	1.00[c]	1.00[c]
	Yes, non hospitalised	3.33 (2.85-3.89)	2.23 (1.98-2.51)	0.95 (0.82-1.09)
	Yes, hospitalised	45.07 (41.02-49.53)	31.31 (28.32-34.62)	5.39 (4.52-6.42)
**All-cause mortality**	No	1.00[c]	1.00[c]	1.00[c]
	Yes, non hospitalised	4.59 (4.22-4.99)	1.33 (1.21-1.46)	0.81 (0.74-0.88)
	Yes, hospitalised	25.23 (23.78-26.77)	11.91 (11.06-12.81)	3.14 (2.84-3.49)
**Cardiovascular disease mortality**	No	1.00[c]	1.00[c]	1.00[c]
	Yes, non hospitalised	4.06 (3.51-4.69)	1.57 (1.36-1.81)	0.87 (0.75-1.00)
	Yes, hospitalised	26.12 (23.83-28.64)	12.12 (10.81-13.60)	3.07 (2.60-3.61)
**Ischemic heart disease mortality**	No	1.00[c]	1.00[c]	1.00[c]
	Yes, non hospitalised	2.00 (1.42-2.82)	1.42 (1.09-1.85)	0.67 (0.51-0.89)
	Yes, hospitalised	9.12 (7.03-11.83)	5.52 (4.13-7.37)	1.96 (1.37-2.81)

a. Defined as a time-varying exposure
b. Adjusted for age, sex, country of birth, income, education, comorbidities and vaccination against Covid-19
c. Reference group
d. Too few events (n≤5)

Supplementary Table 15 Risk of cardiovascular disease and mortality following Covid-19 by risk periods. This table shows the risk of various cardiovascular disease (CVD) and mortality outcomes related to Covid-19 [hazard ratios (HRs) with 95% confidence intervals (CI)] in a cohort of individuals in Sweden aged 40-75 years (n=4,095,414) followed from 1 January 2020 to 31 December 2021, from fully adjusted Cox regression models, by risk periods.

	Ischemic stroke		Intracerebral hemorrhage		Cerebrovascular disease		Acute myocardial infarction	
**Time after Covid-19[a] (days)**	**Events (n)**	**HR[b] (95% CI)**	**Events (n)**	**HR[b] (95% CI)**	**Events (n)**	**HR[b] (95% CI)**	**Events (n)**	**HR[b] (95% CI)**
No Covid-19	14 446	1.00[c]	2 921	1.00[c]	23 857	1.00[c]	16 724	1.00[c]
0-14	128	5.55 (4.66-6.62)	38	7.28 (5.27-10.06)	256	6.68 (5.90-7.56)	101	3.24 (2.67-3.95)
15-30	53	2.09 (1.60-2.74)	13	2.25 (1.30-3.89)	83	1.97 (1.59-2.45)	49	1.44 (1.09-1.91)
31-90	115	1.26 (1.05-1.52)	23	1.10 (0.73-1.67)	186	1.24 (1.07-1.43)	141	1.17 (0.99-1.38)
91-180	146	1.09 (0.92-1.28)	32	1.07 (0.75-1.52)	248	1.13 (0.99-1.28)	220	1.25 (1.10-1.43)
181-365	224	1.06 (0.92-1.21)	51	1.07 (0.81-1.42)	383	1.10 (0.99-1.21)	301	1.05 (0.94-1.18)
366-730	63	1.34 (1.05-1.72)	14	1.35 (0.80-2.29)	104	1.37 (1.13-1.66)	53	0.84 (0.64-1.10)

	Ischemic heart disease		Cardiomyopathy		Heart failure		Deep venous thrombosis	
**Time after Covid-19[a] (days)**	**Events (n)**	**HR[b] (95% CI)**	**Events (n)**	**HR[b] (95% CI)**	**Events (n)**	**HR[b] (95% CI)**	**Events (n)**	**HR[b] (95% CI)**
No Covid-19	37 709	1.00[c]	3 218	1.00[c]	19 597	1.00[c]	13 060	1.00[c]
0-14	339	5.28 (4.74-5.88)	26	3.97 (2.69-5.85)	238	7.77 (6.83-8.84)	184	7.11 (6.14-8.24)
15-30	97	1.38 (1.13-1.68)	13	1.81 (1.05-3.12)	69	2.07 (1.63-2.62)	156	5.46 (4.65-6.40)
31-90	299	1.20 (1.07-1.34)	44	1.70 (1.26-2.30)	179	1.49 (1.29-1.73)	175	1.67 (1.44-1.94)
91-180	440	1.22 (1.11-1.34)	37	0.98 (0.71-1.36)	220	1.23 (1.08-1.41)	189	1.20 (1.04-1.39)
181-365	647	1.10 (1.02-1.19)	80	1.27 (1.01-1.60)	315	1.05 (0.96-1.18)	298	1.21 (1.07-1.36)
366-730	134	1.04 (0.88-1.23)	13	0.97 (0.56-1.67)	98	1.41 (1.15-1.72)	73	1.44 (1.14-1.82)

	Pulmonary embolism		All cause mortality		Cardiovascular disease mortality		Ischemic heart disease mortality	
**Time after Covid-19[a] (days)**	**Events (n)**	**HR[b] (95% CI)**	**Events (n)**	**HR[b] (95% CI)**	**Events (n)**	**HR[b] (95% CI)**	**Events (n)**	**HR[b] (95% CI)**
No Covid-19	11 201	1.00[c]	37 385	1.00[c]	14 737	1.00[c]	4 839	1.00[c]
0-14	918	44.15 (41.12-47.40)	1 271	21.47 (20.28-22.74)	520	23.67 (21.64-25.89)	71	9.32 (7.35-11.81)
15-30	345	15.01 (13.45-16.75)	940	14.77 (13.82-15.78)	374	15.89 (14.31-17.65)	59	7.22 (5.57-9.36)
31-90	218	2.68 (2.34-3.07)	692	3.10 (2.87-3.34)	302	3.66 (3.26-4.11)	39	1.36 (0.99-1.87)
91-180	153	1.29 (1.10-1.52)	417	1.30 (1.18-1.44)	146	1.26 (1.07-1.49)	42	1.07 (0.79-1.46)
181-365	207	1.15 (1.00-1.32)	609	1.14 (1.05-1.24)	229	1.17 (1.03-1.34)	60	0.93 (0.72-1.21)
366-730	38	1.00 (0.73-1.38)	189	1.43 (1.24-1.65)	66	1.32 (1.04-1.68)	15	0.91 (0.54-1.51)

a. Defined as a time-varying exposure
b. Adjusted for age, sex, country of birth, income, education, comorbidities and vaccination against Covid-19
c. Reference group

Supplementary table 16 Risk of three major cardiovascular disease-groups following Covid-19 stratified by social factors, among those without prior comorbidities. This table shows the risk of cerebrovascular disease, ischemic heart disease and thromboembolic disease (deep venous thrombosis and/or pulmonary embolism) respectively, related to Covid-19 [hazard ratios (HRs) with 95% confidence intervals (CI)] in a cohort of individuals in Sweden aged 40-75 years without prior comorbidities (n= 3,214,001) followed from 1 January 2020 to 31 December 2021, from fully adjusted Cox regression models, and stratified by sex, age, income, education and country of birth.

		**Cardiovascular outcomes**		
**Exposure strata**		**Cerebrovascular disease**	**Ischemic heart disease**	**Thromboembolic disease**
**Covid-19**[a]	**Sex**[b]	**HR (95% CI)**	**HR (95% CI)**	**HR (95% CI)**
No	Men	1·00[i]	1·00[i]	1·00[i]
No	Women	0.63 (0.61-0.65)	0.35 (0.34-0.36)	0.78 (0.76-0.81)
Yes	Men	1.45 (1.33-1.59)	1.40 (1.31-1.49)	3.64 (3.43-3.88)
Yes	Women	0.77 (0.68-0.86)	0.42 (0.37-0.47)	1.81 (1.67-1.97)
**Covid-19**[a]	**Age**[c]			
No	40-54 years	1·00[i]	1·00[i]	1·00[i]
No	55-64 years	2.87 (2.73-3.02)	3.60 (3.46-3.75)	1.79 (1.71-1.87)
No	65-75 years	6.53 (6.23-6.84)	7.68 (7.39-7.98)	3.05 (2.93-3.18)
Yes	40-54 years	1.26 (1.10-1.45)	1.23 (1.09-1.38)	2.60 (2.40-2.82)
Yes	55-64 years	3.42 (3.01-3.88)	4.14 (3.76-4.55)	5.18 (4.76-5.64)
Yes	65-75 years	9.95 (8.80-11.25)	11.73 (10.66-12.90)	11.59 (10.56-12.72)
**Covid-19**[a]	**Income**[d]			
No	Low	1·00[i]	1·00[i]	1·00[i]
No	Medium	0.74 (0.71-0.77)	0.95 (0.92-0.98)	0.97 (0.93-1.01)
No	High	0.67 (0.65-0.70)	0.90 (0.87-0.92)	0.95 (0.92-1.00)
Yes	Low	1.46 (1.30-1.65)	1.49 (1.36-1.65)	3.78 (3.46-4.13)
Yes	Medium	0.98 (0.86-1.11)	1.28 (1.17-1.42)	2.92 (2.68-3.17)
Yes	High	0.88 (0.78-1.00)	1.08 (0.98-1.19)	2.52 (2.32-2.74)
**Covid-19**[a]	**Education**[e]			
No	Primary	1·00[i]	1·00[i]	1·00[i]
No	Secondary	0.91 (0.87-0.94)	0.93 (0.90-0.96)	0.98 (0.94-1.03)
No	Tertiary	0.76 (0.73-0.80)	0.72 (0.69-0.74)	0.86 (0.82-0.90)
Yes	Primary	1.48 (1.26-1.73)	1.53 (1.36-1.73)	3.80 (3.39-4.27)
Yes	Secondary	1.19 (1.07-1.33)	1.20 (1.10-1.31)	2.81 (2.60-3.04)
Yes	Tertiary	1.02 (0.89-1.16)	0.91 (0.82-1.01)	2.53 (2.32-2.76)
**Covid-19**[a]	**Country of birth**[f]			
No	Sweden	1·00[i]	1·00[i]	1·00[i]
No	HIC[g]	1.02 (0.96-1.08)	1.18 (1.13-1.24)	0.85 (0.80-0.91)
No	LMIC[h]	0.88 (0.83-0.94)	1.57 (1.51-1.64)	0.60 (0.56-0.64)
Yes	Sweden	1.33 (1.23-1.45)	1.26 (1.18-1.35)	2.73 (2.57-2.89)
Yes	HIC[g]	1.53 (1.19-1.96)	1.78 (1.47-2.16)	3.14 (2.66-3.70)
Yes	LMIC[h]	1.27 (1.07-1.50)	2.41 (2.17-2.68)	3.08 (2.77-3.42)

a. Defined as a time-varying exposure
b. Adjusted for age, country of birth, income, education, and vaccination against Covid-19
c. Adjusted for sex, country of birth, income, education, and vaccination against Covid-19
d. Adjusted for age, sex, country of birth, education, and vaccination against Covid-19
e. Adjusted for age, sex, country of birth, income, and vaccination against Covid-19
f. Adjusted for age, sex, income, education, and vaccination against Covid-19
g. HIC= High income countries
h. LMIC= Low- and middle income countries
i. Reference group

Supplementary table 17 Risk of three major cardiovascular disease-groups following Covid-19 stratified by social factors excluding individuals who immigrated during the five-year lookback period

This table shows the risk of cerebrovascular disease, ischemic heart disease and thromboembolic disease (deep venous thrombosis and/or pulmonary embolism) respectively, related to Covid-19 [hazard ratios (HRs) with 95% confidence intervals (CI)] in a cohort of individuals in Sweden aged 40-75 years (n=3,963,612) followed from 1 January 2020 to 31 December 2021, excluding individuals who immigrated during the five-year lookback period, from fully adjusted Cox regression models, and stratified by sex, age, income, education and country of birth.

Exposure strata		Cerebrovascular disease	Ischemic heart disease	Thromboembolic disease
**Covid-19[a]**	**Sex[b]**	**HR (95% CI)**	**HR (95% CI)**	**HR (95% CI)**
No	Men	1·00[i]	1·00[i]	1·00[i]
No	Women	0.65 (0.64-0.67)	0.39 (0.38-0.40)	0.83 (0.81-0.85)
Yes	Men	1.56 (1.45-1.68)	1.39 (1.32-1.47)	3.43 (3.25-3.61)
Yes	Women	0.82 (0.75-0.91)	0.47 (0.42-0.51)	1.86 (1.74-1.99)
**Covid-19[a]**	**Age[c]**			
No	40-54 years	1·00[i]	1·00[i]	1·00[i]
No	55-64 years	2.79 (2.67-2.92)	3.50 (3.37-3.63)	1.77 (1.70-1.84)
No	65-75 years	6.12 (5.87-6.37)	7.16 (6.92-7.41)	2.77 (2.67-2.86)
Yes	40-54 years	1.31 (1.17-1.48)	1.18 (1.06-1.31)	2.41 (2.24-2.58)
Yes	55-64 years	3.61 (3.25-4.01)	4.12 (3.80-4.47)	4.88 (4.54-5.24)
Yes	65-75 years	9.44 (8.59-10.38)	10.57 (9.78-11.41)	9.51 (8.83-10.24)
**Covid-19[a]**	**Income[d]**			
No	Low	1·00[i]	1·00[i]	1·00[i]
No	Medium	0.75 (0.73-0.78)	0.94 (0.92-0.97)	0.93 (0.90-0.97)
No	High	0.68 (0.66-0.70)	0.89 (0.86-0.91)	0.89 (0.86-0.92)
Yes	Low	1.55 (1.41-1.70)	1.44 (1.33-1.55)	3.21 (2.99-3.45)
Yes	Medium	1.02 (0.92-1.14)	1.27 (1.17-1.38)	2.63 (2.45-2.81)
Yes	High	0.94 (0.85-1.04)	1.09 (1.00-1.18)	2.36 (2.20-2.54)
**Covid-19[a]**	**Education[e]**			
No	Primary	1·00[i]	1·00[i]	1·00[i]
No	Secondary	0.92 (0.89-0.95)	0.93 (0.91-0.96)	0.98 (0.95-1.01)
No	Tertiary	0.78 (0.76-0.81)	0.72 (0.70-0.74)	0.86 (0.83-0.89)
Yes	Primary	1.57 (1.38-1.77)	1.46 (1.33-1.61)	3.42 (3.11-3.76)
Yes	Secondary	1.29 (1.18-1.41)	1.18 (1.10-1.27)	2.71 (2.54-2.89)
Yes	Tertiary	1.09 (0.98-1.21)	0.97 (0.89-1.06)	2.35 (2.18-2.53)
**Covid-19[a]**	**Country of birth[f]**			
No	Sweden	1·00[i]	1·00[i]	1·00[i]
No	HIC[g]	1.03 (0.99-1.08)	1.20 (1.16-1.25)	0.87 (0.83-0.92)
No	LMIC[h]	0.85 (0.81-0.90)	1.46 (1.41-1.51)	0.63 (0.59-0.66)
Yes	Sweden	1.40 (1.30-1.49)	1.29 (1.22-1.37)	2.65 (2.53-2.78)
Yes	HIC[g]	1.56 (1.28-1.91)	1.66 (1.41-1.96)	2.79 (2.42-3.22)
Yes	LMIC[h]	1.36 (1.19-1.56)	2.12 (1.93-2.33)	2.72 (2.48-2.98)

a. Defined as a time-varying exposure
b. Adjusted for age, country of birth, income, education, comorbidities and vaccination against Covid-19
c. Adjusted for sex, country of birth, income, education, comorbidities and vaccination against Covid-19
d. Adjusted for age, sex, country of birth, education, comorbidities and vaccination against Covid-19
e. Adjusted for age, sex, country of birth, income, comorbidities and vaccination against Covid-19
f. Adjusted for age, sex, income, education, comorbidities and vaccination against Covid-19
g. HIC= High income countries
h. LMIC= Low- and middle income countries
i. Reference group

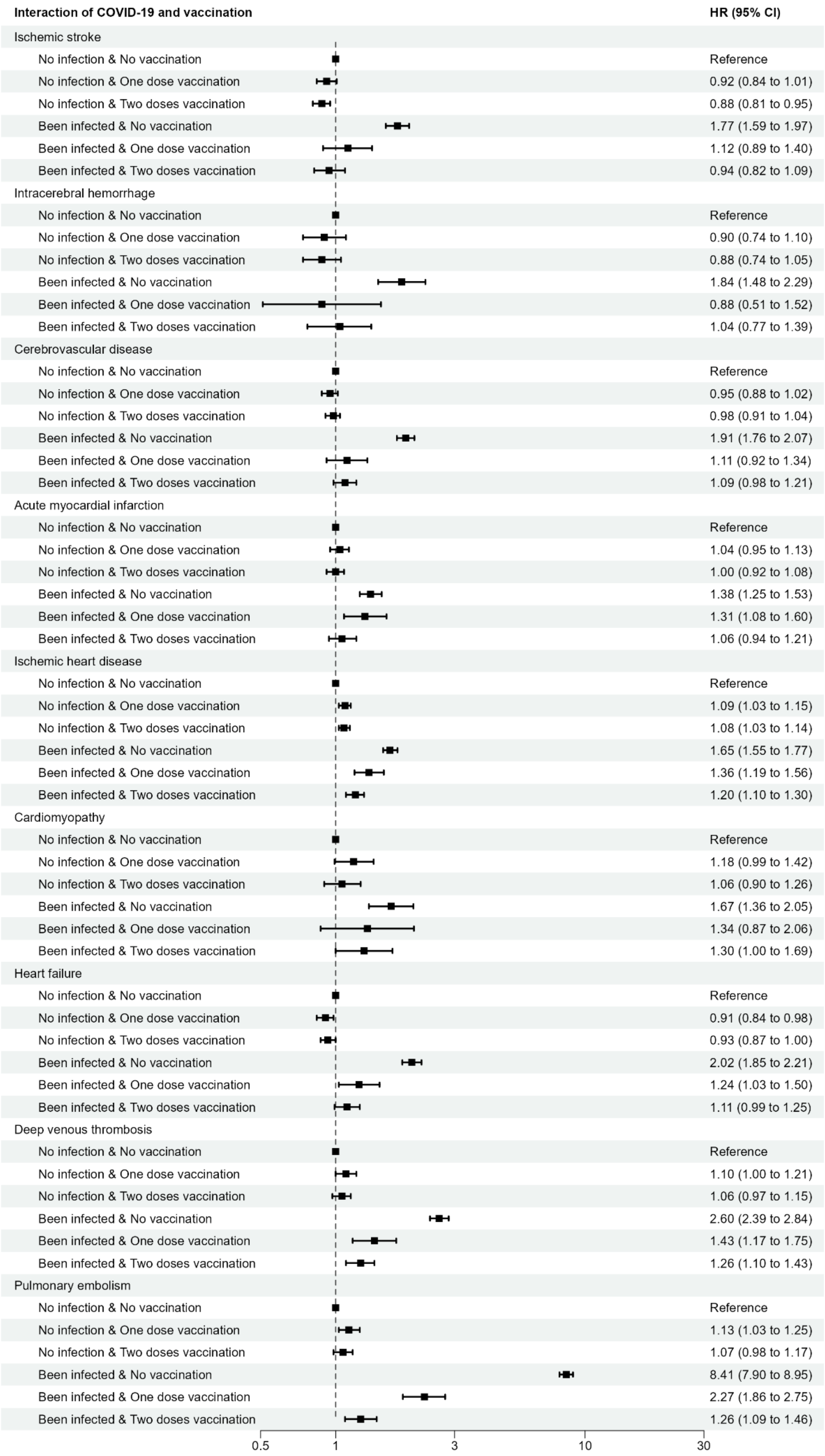
Interaction of COVID-19 and vaccination
HR (95% CI)
Ischemic stroke
No infection & No vaccination
Reference
No infection & One dose vaccination
0.92 (0.84 to 1.01)
No infection & Two doses vaccination
0.88 (0.81 to 0.95)
Been infected & No vaccination
1.77 (1.59 to 1.97)
Been infected & One dose vaccination
1.12 (0.89 to 1.40)
Been infected & Two doses vaccination
0.94 (0.82 to 1.09)
Intracerebral hemorrhage
No infection & No vaccination
Reference
No infection & One dose vaccination
0.90 (0.74 to 1.10)
No infection & Two doses vaccination
0.88 (0.74 to 1.05)
Been infected & No vaccination
1.84 (1.48 to 2.29)
Been infected & One dose vaccination
0.88 (0.51 to 1.52)
Been infected & Two doses vaccination
1.04 (0.77 to 1.39)
Cerebrovascular disease
No infection & No vaccination
Reference
No infection & One dose vaccination
0.95 (0.88 to 1.02)
No infection & Two doses vaccination
0.98 (0.91 to 1.04)
Been infected & No vaccination
1.91 (1.76 to 2.07)
Been infected & One dose vaccination
1.11 (0.92 to 1.34)
Been infected & Two doses vaccination
1.09 (0.98 to 1.21)
Acute myocardial infarction
No infection & No vaccination
Reference
No infection & One dose vaccination
1.04 (0.95 to 1.13)
No infection & Two doses vaccination
1.00 (0.92 to 1.08)
Been infected & No vaccination
1.38 (1.25 to 1.53)
Been infected & One dose vaccination
1.31 (1.08 to 1.60)
Been infected & Two doses vaccination
1.06 (0.94 to 1.21)
Ischemic heart disease
No infection & No vaccination
Reference
No infection & One dose vaccination
1.09 (1.03 to 1.15)
No infection & Two doses vaccination
1.08 (1.03 to 1.14)
Been infected & No vaccination
1.65 (1.55 to 1.77)
Been infected & One dose vaccination
1.36 (1.19 to 1.56)
Been infected & Two doses vaccination
1.20 (1.10 to 1.30)
Cardiomyopathy
No infection & No vaccination
Reference
No infection & One dose vaccination
1.18 (0.99 to 1.42)
No infection & Two doses vaccination
1.06 (0.90 to 1.26)
Been infected & No vaccination
1.67 (1.36 to 2.05)
Been infected & One dose vaccination
1.34 (0.87 to 2.06)
Been infected & Two doses vaccination
1.30 (1.00 to 1.69)
Heart failure
No infection & No vaccination
Reference
No infection & One dose vaccination
0.91 (0.84 to 0.98)
No infection & Two doses vaccination
0.93 (0.87 to 1.00)
Been infected & No vaccination
2.02 (1.85 to 2.21)
Been infected & One dose vaccination
1.24 (1.03 to 1.50)
Been infected & Two doses vaccination
1.11 (0.99 to 1.25)
Deep venous thrombosis
No infection & No vaccination
Reference
No infection & One dose vaccination
1.10 (1.00 to 1.21)
No infection & Two doses vaccination
1.06 (0.97 to 1.15)
Been infected & No vaccination
2.60 (2.39 to 2.84)
Been infected & One dose vaccination
1.43 (1.17 to 1.75)
Been infected & Two doses vaccination
1.26 (1.10 to 1.43)
Pulmonary embolism
No infection & No vaccination
Reference
No infection & One dose vaccination
1.13 (1.03 to 1.25)
No infection & Two doses vaccination
1.07 (0.98 to 1.17)
Been infected & No vaccination
8.41 (7.90 to 8.95)
Been infected & One dose vaccination
2.27 (1.86 to 2.75)
Been infected & Two doses vaccination
1.26 (1.09 to 1.46)
0.5
1
3
10
30

Supplementary Figure 1 Interaction analyses by Covid-19 and vaccination This figure shows the risk of various cardiovascular disease (CVD) outcomes related to Covid-19 [hazard ratios (HRs) with 95% confidence intervals (CI)] in a cohort of individuals in Sweden aged 40-75 years (n=4,095,414) followed from 1 January 2020 to 31 December 2021, from Cox models with full adjustments for sociodemographic factors and comorbidities, by Covid-19 and vaccination**.**
